# Correlation of long non-coding RNA *H19* expression with cisplatin-resistance and clinical outcome in lung adenocarcinoma

**DOI:** 10.18632/oncotarget.13708

**Published:** 2016-11-29

**Authors:** Qi Wang, Ningning Cheng, Xuefei Li, Hui Pan, Chunyu Li, Shengxiang Ren, Chunxia Su, Weijing Cai, Chao Zhao, Limin Zhang, Caicun Zhou

**Affiliations:** ^1^ Department of Medical Oncology, Shanghai Pulmonary Hospital, Tongji University, Tongji University Medical School Cancer Institute, Shanghai, 200433, P.R. China; ^2^ Department of Radiation Oncology, Shanghai General Hospital, Shanghai Jiaotong University, Shanghai, 201620, P. R. China; ^3^ Department of Lung Cancer and Immunology, Shanghai Pulmonary Hospital, Tongji University, Tongji University Medical School Cancer Institute, Shanghai, 200433, P.R. China; ^4^ International Medical School, Tianjin Medical University, Tianjin, 300070, P.R. China

**Keywords:** long non-coding RNA, lung adenocarcinoma, H19, cisplatin, A549/DDP cells

## Abstract

The acquired drug resistance would influence the efficacy of cisplatin-based chemotherapy in non-small-cell lung cancer. The present study aimed to investigate the correlation of long non-coding RNA (lncRNA) *H19* with cisplatin-resistance and clinical outcome in lung adenocarcinoma. In our study, the expression of *H19* in cisplatin-resistant A549/DDP cells was unregulated. Knockdown of *H19* restored the response of A549/DDP cells to cisplatin. *H19*-mediated chemosensitivity enhancement was associated with metastasis, induction of G0/G1 cell-cycle arrest, cell proliferation, and increased apoptosis. Furthermore, lncRNA *H19* expression was significantly related to TNM stage and metastasis (*P* = 0.012). Overexpression of *H19* was negatively correlated with cisplatin-based chemotherapy response in patients. Patients with high *H19* expression exhibited a significantly shorter median progression-free survival (PFS) [4.7 months] than the low-expression patients (6.3months) [*P* = 0.002]. In summary, *H19*-mediated regulation of cisplatin resistance in human lung adenocarcinoma cells is demonstrated for the first time. *H19* could potentially serve as a molecular marker to predict the clinical outcomes of lung adenocarcinoma patients.

## INTRODUCTION

Lung cancer is one of the most common fatal cancers worldwide, with a 5-year survival as low as 13% [1, 2]. Non-small cell lung cancer (NSCLC) accounts for 80 – 85% of all the lung cancers [3]. Currently, most standard initial treatment strategies for advanced NSCLC involve platinum-based doublet chemotherapy with the use of cisplatin as an effective cytotoxic agent in combination with other agents [4, 5]. However, the efficacy of cisplatin treatment would be impaired by emergence of chemoresistance [6]. Therefore, further elucidation of molecular mechanisms underlying chemoresistance would promote our understanding of NSCLC treatment failure and development.

Long non-coding RNAs (LncRNAs) stand for a new class of non-protein-coding RNAs. They are typically longer than 200 nt and do not function as templates for protein synthesis [7–10]. It has been shown that altered lncRNA would lead to aberrant expression of gene products, which will lead to different disease condition, cancer included [11, 12]. LncRNA-*H19* gene represents to be one of the first group of genes, which has proved to be maternally expressed and paternally imprinted. Accumulating evidence indicates that *H19* gene is an oncogenic lncRNA in bladder and hepatocellular carcinoma and breast cancer [13–19]. While *H19* exhibits oncogenic functions in some types of cancer, it also acts as a tumor suppressor [20, 21], depending on the type of cancer and cellular context. Thus, the discovery of lncRNA *H19* may be ascribed a major role in chemoresistance in cancer cells [22]; the mechanism underlying NSCLC is yet unclear.

In the current study, we observed the potential mechanisms, biological function and clinical feature of lncRNA *H19* in lung adenocarcinoma. Combined together, this research studies the potential of *H19*, taking it as a valid therapeutic target for the cisplatin resistance reversal in patients suffering from lung adenocarcinoma.

## RESULTS

### Overexpression of *H19* was correlated with acquired resistance to cisplatin

**Figure 1:**
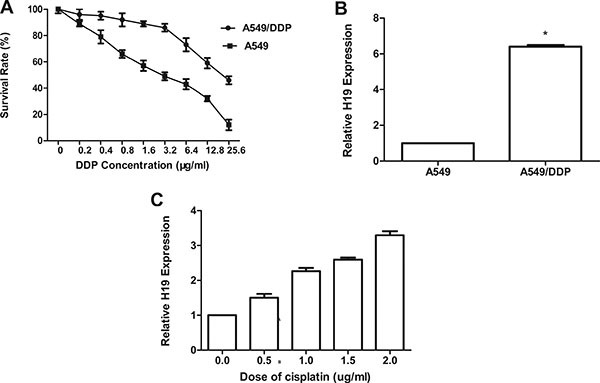
(**A**) The sensitivity to cisplatin of A549 and A549/DDP was detected by CCK-8 (Cell Counting kit-8). Cells were exposed to various concentrations of cisplatin for 48 h. (**B**) The expression of H19 in A549/DDP was significantly higher than that in A549. (**C**) A549/DDP cells were cultured with various concentrations of cisplatin for 48 h; qRT-PCR was performed to detect H19 expression. Every experiment was conducted at least three times, and the average is shown (mean ± SD).

The CCK-8 assay is used to test the cisplatin sensitivity. As shown in Figure 1A, the IC50 of cisplatin in the cell line of drug-resistant A549/DDP was about 17.06 ± 0.23 μg/mL. This was 3.4 folder higher compared with the cell line of A549, which is 5.02 ± 0.28 μg/mL. Thus, A549/DDP cells showed increased resistance against cisplatin compared with parental cells. To further ascertain whether *H19* plays an important role in the acquired cisplatin resistance of lung adenocarcinoma cells. The qRT-PCR assay was used to examine the H19 expression in A549/DDP cells and was detected to be dramatically increased almost about 6.3-fold (*P* < 0.01; Figure 1B). In the case of parental A549 cells being treated with different concentration of cisplatin, qRT-PCR showed a dramatic increase in the *H19* expression (Figure 1C). Therefore, the growing expression *H19* level in adenocarcinoma cells would respond to cisplatin treatment.

### The cisplatin sensivitiy in cisplatin resistant human lung adenocarcinoma cell line was restored by *H19* inhibition (A549/DDP)

**Figure 2:**
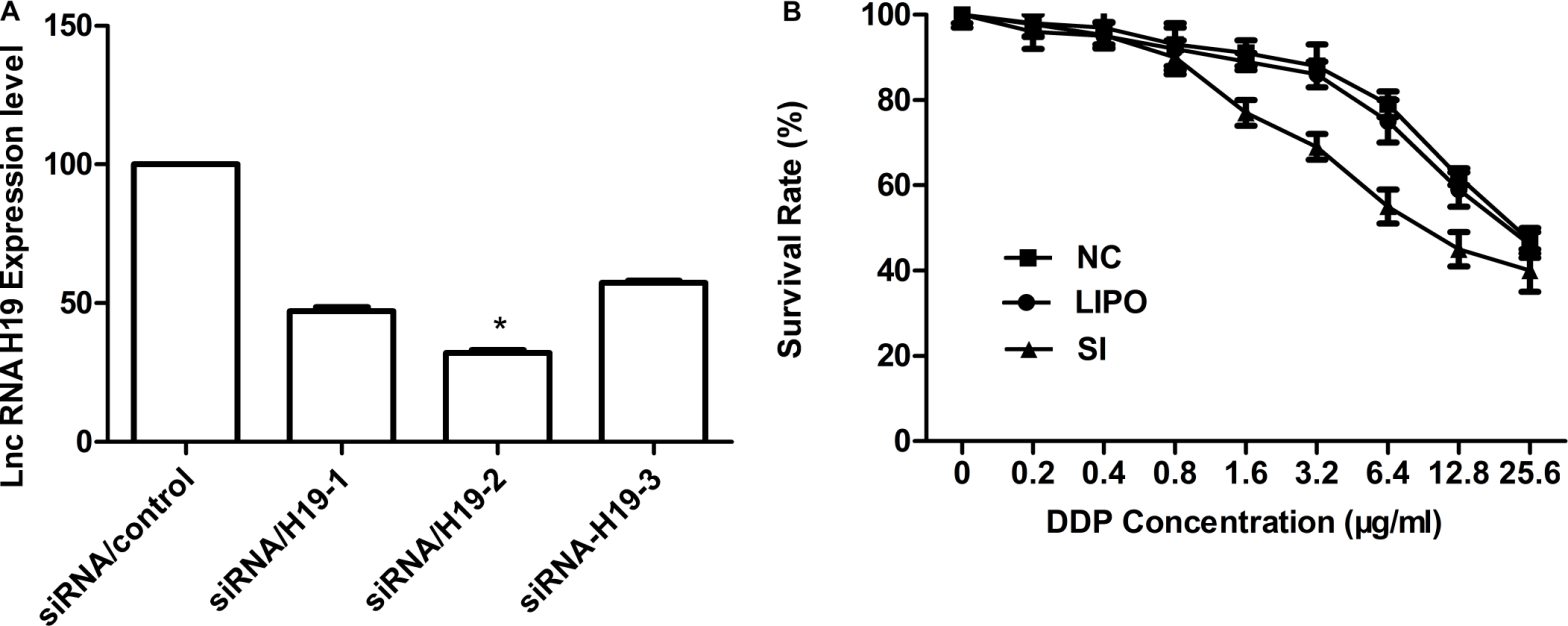
(**A**) qRT-PCR detection of H19 expression in A549/DDP cells after silencing of H19 by siRNA. The relative expression of H19 was 66.6% lower with si-H19-2 than with the negative control. (**B**) A549 sensitivity to cisplatin was detected by CCK-8 (Cell Counting Kit-8). Cells were exposed to various doses of cisplatin for 48 h. Inhibiting the H19 gene resulted in an approximately 47.12% decrease in the cisplatin IC50 in A549/DDP cells (IC50 in si-H19-2 and A549/DDP cells, 8.13 and 24.1 μg/mL, respectively).

To assess the function of *H19* in acquired cisplatin-resistant A549/DDP cells, the silencing capacity of si-H19-2 was evaluated by qRT-PCR. Si-H19-2 showed an optimal gene-silencing effect in comparison with si-H19-1 and the negative control (NC) (*P* < 0.01) [Figure 2A]. Thus, siRNA/H19-2 was utilized in the subsequent experiments. Then, the CCK-8 assay was used to examine the effect of *H19* expression on IC50 of cisplatin to A549/DDP cells. The outcomes revealed that siRNA/H19 would decrease the IC50 of cisplatin on A549/DDP cells significantly with a rate of 47.12%, and the sensitivity to cisplatin was partially restored (*P* < 0.05; Figure 2B). Thus, *H19* may play a vital role in cisplatin resistance in lung adenocarcinoma cancer.

### Downregulation of *H19* expression affected cell apoptosis, cell cycle, and cell migration

**Figure 3:**
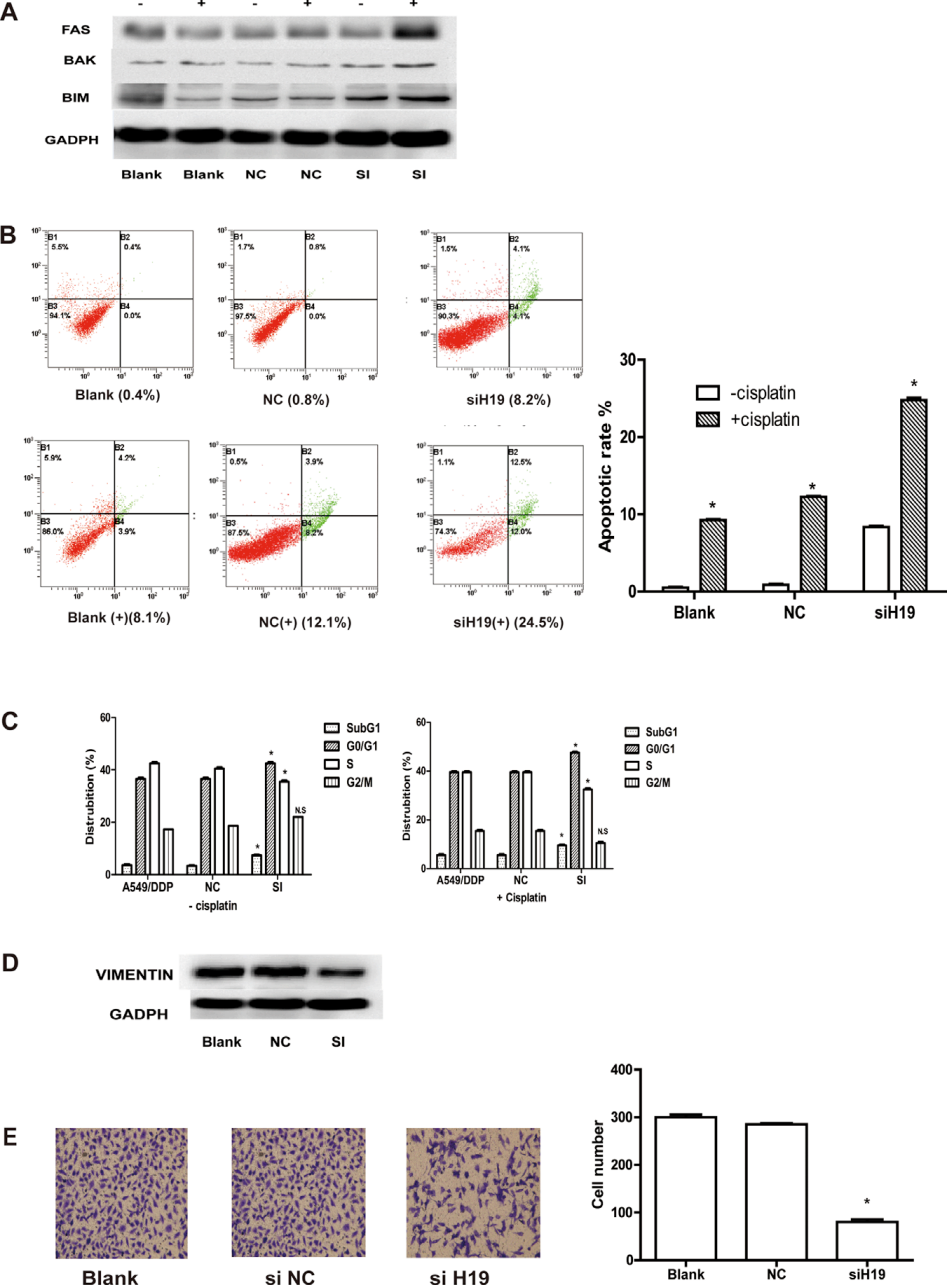
(**A**) Western blot analysis: FAS, BAK, and BIM (Bcl-2 interacting mediator of cell death). “+” indicates apoptosis markers detected with cisplatin, “−” indicates apoptosis detected without cisplatin. (**B**) Cisplatin-induced apoptosis in A549/DDP cells was demonstrated by flow cytometric analysis. Cells were treated with cisplatin for 48 h and then analyzed for early apoptotic cells (bottom right quadrant) and late apoptotic cells (top right quadrant). The percentage of cells in the two quadrants are shown. “+” stands for cisplatin-induced apoptosis in A549/DDP cells, “−” stands for apoptosis in A549/DDP cells without cisplatin. (**C**) Flow cytometry analysis of cell cycle distribution in si-H19-2-treated A549/DDP cells combined with various concentrations of cisplatin (0, 1.0 or 2.0) si-NC-treated and non-treated A549/DDP cells. NS indicates *P* > 0.05 and *indicates *P* < 0.05; respectively. (**D**) Western blot analysis of the proteins of epithelial-mesenchymal transition (EMT) in si-H19-2 treated and negative control (NC)-treated A549/DDP cells and non-treated A549/DDP cells. (**E**) Representative images of migratory cells on the membrane (magnification,100×).

As refractoriness to apoptosis induced by cisplatin is one of the major features of resistance to chemotherapy in NSCLC [23], the effect of *H19* on cell apoptosis was examined. We observed that the expressions of FAS, BAK, and BAX (the activation of which may be involved in cell apoptosis) were increased post transfection by si-H19-2 (Figure 3A). However, other apoptosis markers such as BAD, caspases3, and caspase8 did not alter significantly (Supplementary Figure S1). A considerably higher percentage of apoptotic cells were found in si-H19-2 treated cells (24.5%) in comparison with those transfected with negative control (12.1%) and blank group (8.1%) (Figure 3B). Moreover, we found that the percentage of si-H19-2 A549/DDP cells contained in G0/G1 and subG1 phases in cell cycle increased while the percentage of S phase cells reduced with the growing cisplatin doses (Figure 3C). These results indicate that inhibition of *H19* induces apoptosis in cells resistant to cisplatin. Since epithelial-mesenchymal transition (EMT) plays a critical role in resistance to cisplatin-resistant, an increase of mesenchymal markers such as Vimentin [24, 25] determines the relationship between *H19* and EMT markers. The data in Figure 3D indicated that knockdown of *H19* enhanced the expression of Vimentin. The down-regulation of *H19* also significantly decreased cell migration, as what is confirmed via quantitative analysis by a transwell system. The average proportion of the siH19-treated cell lines was decreased down to 26.7 ± 1.31% (*P* < 0.05) and 95.2 ± 0.63% in the NC cells (*P* > 0.05), compared with the blank control (Figure 3E). To sum up, these findings indicate that *H19* expression may active cell apoptosis and migration to promote resistance to cisplatin.

### Impact of overexpression of *H19* on pathological characteristics and PFS in NSCLC patients receiving cisplatin therapy

**Figure 4:**
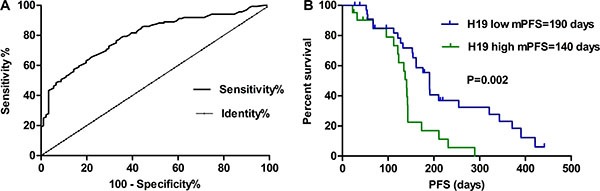
(**A**) Using a cut-off **value** 1.178 ng/mL, ROC analysis revealed an area under the curve (AUC) of 0.751 (*P* < 0.01), indicating a diagnostic sensitivity of 72.79% and specificity of 70.33% to differentiate high or low H19 expression levels from NSCLC patients. (**B**) Survival curves were analyzed by log-rank test and Kaplan-Meier method. Patients with low H19 levels had a dramatically longer survival than those with high H19 levels (*P* = 0.002).

As shown in Table 1, the *H19* expression level in tissues was closely correlated with the metastasis (*P* = 0.012) and TNM stage (*P* = 0.012). However, no significant correlation was found between other clinicopathological parameters (including family history, pathological types, smoking history, gender and age) and *H19* expression. ROC curve analysis established the optimal cut-off value 1.178 of *H19* expression level in 136 adenocarcinoma tissues, which yielded an area under the curve (AUC) of 0.751 (*P* < 0.01). Therefore, *H19* achieved a diagnostic sensitivity of 72.79% and specificity of 70.33% (Figure 4A). The cut-off value of 1.178 ng/mL of *H19* was chosen to categorize patients as low *H19* (*n* = 69) or high *H19* (*n* = 77). According to the Kaplan-Meier survival curve analysis, patients with low *H19* have longer median PFS (6.3 months; 95% CI = 0.1912–1.282) compared with those with high expression, which is about (4.7 months; 95% = 1.516–6.709) [Figure 4B]. As shown in Table 2, the Cox proportional hazards analysis was adopted to further assess the expression level of *H19* as a prognostic biomarker. According to the univariate analysis, the expression level of *H19* was associated with prognosis (HR 2.384; *P* = 0.008) while the multivariate analysis significantly associated high *H19* to a shorter PFS (HR 2.224; *P* = 0.016). As expected, it is found that the disease stage was closely associated with decreased PFS in both univariate and multivariate analysis (*P* < 0.05).

**Table 1:** Relation of tissue H19 to clinicopathological characteristics

Characteristics	Number	Percentage	*H19*levels	*P* value
Gender				0.983
Male	100	73.5%	0.114	
Female	36	26.5%	0.116	
Age				0.676
< 60	70	51.5%	0.128	
> = 60	66	48.5%	0.101	
Lymph nodes				0.378
N0-1	34	25%	0.067	
N2-3	102	75%	0.131	
Metastasis				0.012
M0	53	38.9%	0.018	
M1	83	61.1%	0.176	
Clinical stage				0.012
IIIB	53	38.9%	0.018	
IV	83	61.1%	0.176	
Smoking history				0.474
Yes	90	66.2%	0.146	
Never	46	33.8%	0.989	
Family history				0.262
Yes	14	10.3%	0.218	
No	122	89.7%	0.103	

**Table 2:** Univariate and multivariate analyses of H19 status with regard to PFS

Variables	HR	95%CI	*P* value
Univariate analysis			
H19 (low vs. high)	2.384	1.257–4.520	0.008
Age(≥ 60 vs. < 60)	0.966	0.509–1.833	0.917
Gender(male vs. female)	0.882	0.474–1.644	0.693
Stage(IIIB vs. IV)	3.167	1.333–7.527	0.009
N(0–1 vs. 2–3)	1.210	0.595–2.460	0.599
M(yes vs. no)	3.167	1.333–7.527	0.009
Smoking history	0.631	0.342–1.167	0.142
Family history	0.907	0.123–6.665	0.923
Multivariate analysis			
H19 (low vs. high)	2.224	1.163–4.253	0.016
Age (≥ 60 vs. < 60)	0.647	0.346–1.209	0.172
Gender(male vs. female)	1.425	0.705–2.880	0.324
Stage(IIIB vs.IV)	2.988	1.250–7.143	0.014
N(0–1 vs. 2–3)	1.206	0.464–2.272	0.949
M(yes vs. no)	2.988	1.250–7.143	0.014
Smoking history	0.648	0.347–1.211	0.174.
Family history	2.359	0.298–18.703	0.417

CI, confidence interval; HR, hazard ratio; N, Lymph Nodes; M, Metastasis.

### *H19* expression in lung adenocarcinoma tissues is correlated with the patients’ clinical response to cisplatin-based chemotherapy

To have a better understanding of the *H19* expression in lung adenocarcinoma tissues and the clinical response to cisplatin-based regimens, qRT-PCR was used to examine the *H19* levels on tumor tissues among 136 eligible patients treated with cisplatin-based chemotherapy. We analyzed patient responses to platinum-based chemotherapy (Table 3). Patients with high *H19* expression (≥ 1.178 ng/mL), platinum-based chemotherapy was significantly less effective than in those with low *H19* expression (< 1.178 ng/mL) [*P* = 0.015; *P* = 0.034, respectively]. Thus, to conclude, *H19* expression was negatively correlated with patients’ response to platinum-based chemotherapy.

**Table 3:** Association between tissue H19 level and cisplatin based chemotherapy efficacy

	High H19 expression (≥ 1.178 ng/ml)	Low H19 expression (< 1.178 ng/ml)	*P*value
PR, n	16	22	
SD, n	20	14	
PD, n	41	33	
ORR, %	21.3	31.6	0.015
DCR, %	39.7	51.4	0.034

ORR, objective response rate; DCR, disease control rate; PR, partial response; PD, progression disease; SD, stable disease.

## DISCUSSION

Acquired resistance is one of the major barriers coming across in cancer chemotherapy. Many studies have illustrated the substaintial epigenetic alterations contained in drug-resistant lung cancer cells though evidence about the genetic alteration following chemotherapeutic treatment is constrained [26, 27]. Some studies have also demonstrated the role of lncRNAs in chemoresistance regulation [28–30]. However, the general contribution of lncRNAs to cisplatin resistance keeps unknown largely. Therefore, we explored the underlying molecular mechanism of lncRNAs in acquired resistance to cisplatin in NSCLC.

*H19* was the first imprinting lncRNA, expressed in the maternal allele rather than paternal and is transcribed for the *H19*/IGF2 gene cluster existed in human chromosome 11p15.5 [31, 32]. *H19* is demonstrated to be correlated with drug resistance in liver, breast, and bladder cancer [33–35]. Herein, in order to obtain the insight into the molecular mechanisms of *H19* in cisplatin resistance in NSCLC, we made a comparison between cisplatin-resistant and cisplatin-sensitive human lung cancer cells by qRT-PCR and found that *H19* is upregulated in cisplatin-resistant cells. Therefore, *H19* may be suggested as an oncogenic factor in cisplatin resistance of NSCLC.

To our knowledge, this is the first report to link *H19* gene with lung adenocarcinoma cisplatin resistance. We observed that *H19* knockdown could partially restore the sensitivity of A549/DDP cells. Based on our clinical data, it can be inferred that the lncRNA *H19* expression was significantly connected with the metastasis and TNM stage. Patients with high *H19* level are strongly negatively associated with the patients’ response to cisplatin-based chemotherapy. Patients with low *H19* expression have dramatically longer median PFS than those with high expression. The univariate and multivariate analysis of PFS revealed that *H19* and tumor stage were independent prognostic factors. The clinical data were consistent with the cell experiments and further confirmed our hypothesis. The present results were similar to those of other lncRNAs associated with cancer prognosis. *HOTAIR* expression from patients diagnosed with stage IV colorectal cancer (CRC) was demonstrated to associated with poor prognosis [36, 37]. *MALAT1* (metastasis associated lung adenocarcinoma transcript 1) has also been associated with the poor prognosis and metastasis NSCLC patients [38]. Additionally, *H19* is correlated with poor prognosis and is upregulated in gastric cancer [21, 39, 40].

Notably, the chemoresistance of bladder cancer cells is increased by *UCA1* through regulating the Wnt signaling [41]. *HOTAIR*, has been proved to be upregulated in lung cancer. Besides, it also regulated the chemoresistance via the modulation of cell cycle and apoptosis [28]. Our study indicated that the siRNA *H19* increasing the sensitivity of lung adenocarcinoma cells to cisplatin might be correlated with G0/G1 cell cycle arrest, apoptosis enhancement and migration. We found that the knockdown of *H19* was related to apoptosis proteins FAS, BAX, and BAK. Consecutively, other researches have illustrated that lncRNA *H19* was actively associated with the E2F1 (E2F transcription factor 1) to promote the cell-cycle progression of breast cancer cells [42]. In addition, the effects of *H19* on cell proliferation and invasiveness in our study were similar to those of *H19* promoting pancreatic cancer metastasis. It is achieved by suppressing Let-7 over target HMGA2-mediated EMT [43]. These studies substantiate our results.

Therefore, we consider that *H19* may play a major role in overcoming acquired resistance to cisplatin as a novel epigenetic regulator in lung adenocarcinoma such as *ERCC1* [44], and sensitizing *H19* might be an efficient therapeutic intervention in cisplatin resistance of lung adenocarcinoma. However, further investigations are imperative in order to elucidate the mechanisms *H19*-mediated regulatory pathway of migration and cisplatin resistance. Furthermore, these data will lay the foundation to explore the related genes’ expression, their regulation, and function. These studies will provide insight for the improvement of the clinical treatment and prediction of NSCLC patients.

## MATERIALS AND METHODS

### Cell lines and cell culture

The human lung adenocarcinoma cell line A549 and cisplatin-resistant variant cell line A549/DDP (obtained from Shanghai Pulmonary Hospital) were cultured in DMEM medium (Life Technologies). It is supplemented with 100 U/mL penicillin in a humid incubator which contained 5% CO_2_ at 37°C and also 10% fetal calf serum (Gibco, Gran Island, NY, USA). Moreover, 2 mg/L cisplatin was contained in the A549/DDP cell medium to keep its drug-resistant phenotype. The subsequent experiments will use cells in the logarithmic phase of growth.

### Patients and tissue samples

A total of 136 tumor tissue samples were harvested from advanced lung adenocarcinoma patients who treated with cisplatin-based chemotherapy at Shanghai Pulmonary Hospital during January 2011 to November 2013. The patient tissues were immediately frozen in liquid nitrogen and were stored at −80 °C until further use. The following criteria were to be fulfilled for inclusion in the study: patients who suffered from primary lung adenocarcinoma; a clinical stage of IIIB to IV; the histological diagnosis of lung adenocarcinoma with more than one measurable lesion; a histological diagnosis of lung adecnocarcinoma with at least one measurable lesion; first line chemotherapy with cisplatin 75 mg/m^2^ on day 1 and gemcitabine 1250 mg/m^2^ on days 1 and 8 or pemetrexed 500 mg/m^2^ on day 1 every 21 days for maximum of 4 cycles [45].Tumor staging was determined basd on the seventh editon of TNM categorizing of International Union Against Cancer. Ethics Committee of Shanghai Pulmonary Hospital approved this research. Each study participant provided the written informed consent.

### Transfection of siRNA

To assess the lncRNA *H19* inhibition, 50 nM of lncRNA *H19* siRNA (GuangZhou RIBO, China) were transfected into A549/DDP cells by Lipofectamine 2000 based on manufacturer's instructions. Cells transfected with the scrambled siRNA have been adopted as the negative control. The cells were collected 48 h after the transfection. Three pairs of siRNA named siRNA H19-1, siRNA H19-2 and siRNA H19-3. Compared with control, the expression level of lncRNA *H19* was only decreased successfully by si-H19-2. The target sequence of si-H19-2 was listed as follows: 3'- dTdTGGAGAUCGAACCUUUACUU-5', the antisense strand, 5'- CCUCUAGCUUGGAAAUGAAdTdT-3' and sense strand.

### RNA extraction and quantitative real-time PCR

Total RNA was extracted from tissues using an RNeasy MINI Kit (QIAGEN) or cell lines using the TRIzol reagent (Invitrogen, USA). Coming to qRT-PCR assay, RNA was reverse transcribed to cDNA from 1.0 μg total RNA through a Reverse Transcription Kit (Takara, Japan). Real-time PCR (RT-PCR) analyses were conducted using Power SYBER Green (Takara, Shiga, Japan). All protocols have been carried out based on the instructions offered by manufacturer. *H19* primers were designed by Sangon Biotech (China). Glyceraldehyde 3-phosphate dehydrogenase (GADPH) was used as an endogenous control. ABI 7500 Fast Real-Time PCR System (Applied Biosystems, CA, USA) was adopted to perform the qRT-PCR assays.

### *In vitro* chemosensitivity assay

The *in vitro* chemosensitivity of cisplatin-resistant and parental A549 cells to cisplatin was determined by CCK-8 assay. Briefly, cells have been seeded into 96-well plates (5 × 10^3^ cells/well) and hence to make it possible for overnight adherence. Subsequently, the cells were treated with various concentrations (0, 0.1, 0.2, 0.4, 0.8, 1.6, 3.2, 6.4, 12.8, and 25.6 μg/mL) of cisplatin. 10 μL of CCK-8 (Cell Counting Kit-8, C04-13; Dojindo Laboratories, Kumamoto, Japan) was put into each well after 48 h and was incubated for 4 h under 37°C. A micro-plate reader at 450 nM has been used to analyze the plates. Every experiment was done more than three times.

### Western blot assay

The siRNAs were transfected into the cells. Whole protein lysates were extracted from the cells with RIPA lysis buffer (KenGEN, China) and quantified by BCA Protein Assay Kit (Beyotime, China). Then, 30 μg lysates were resolved by SDS-PAGE and transferred to PVDF membranes (Millipore, USA). The membrane was blocked with 5% nonfat milk. Then, it was incubated with primary antibodies against BAK, BAX, FAS and Vimentin, followed by incubation with a horseradish peroxidase-conjugated secondary antibody (Santa Cruz; USA). GADPH served as the loading control (CST, USA).

The membrane was blocked with 5% nonfat milk and incubated with primary antibodies against FAS, BAX, BAK, and Vimentin, followed by incubation with a horseradish peroxidase-conjugated secondary antibody (Santa Cruz; USA).

### Flow cytometric analysis of cell cycle and apoptosis

Cells were plated in 6-well plates (2 × 10^5^ cells/well). 24 h post transfection of siRNA H19-2 as described above, A549/DDP cells were treated by DDP at a final concentration of 5 mg/L. The propidium iodide stained the cells after 24 h. The BD Cycle Test Plus DNA Reagent Kit (BD Biosciences, Shanghai, China) has been used in the cell-cycle analysis, following the protocol offered by the manufacturer. The cells were analyzed by FAC scan (BD Biosciences, Shanghai, China), and the percentage of cells in G0/G1, S, or G2/M phase was estimated. Every experiment was conducted at least three times.

The A549/DDP cells were seeded in 6-well plates. Afterward, they were transfected through negative control and si-H19-2. These cells were resuspended in binding buffer, washed with PBS twice and trypsinized after 48 h. Then, Annexin V/PI (Invitrogen, USA) was used to stain the cells for 15 min in the dark at the room temperature. Then, cell population analysis was conducted by flow cytometry.

### Cell migration assays

Coming to the migration assays, at 48 h post-transfection, 5 × 10^4^ cells in serum-free media were put into the upper chamber (8 μm pore size; Millipore) of the insert, the lower chamber was added with medium containing 10% FBS. After 24 h-incubation, the cells contained in the upper membrane were removed by cotton wool. The cells that migrated through the membrane were stained with methanol and 0.1% crystal violet, imaged, and counted using an IX7 inverted microscope (Olympus, Tokyo, Japan). Experiments were conducted three times independently.

### Statistical analysis

All statistics are presented as means ± SE. They were analyzed through Prism 5.0 software (GraphPad). Mann-Whitney *U* test, one-way ANOVA and Student's *t-test* (2-tailed) were used to detect the *in vitro* data using SPSS 17.0 software (IBM, IL, USA). *P* < 0.05 was deemed as statistically significant (*P* < 0.05).

## SUPPLEMENTARY MATERIALS FIGURES



## Abbreviations

AUC: Area under the curve
CCK-8: cell counting kit-8
EMT: epithelial-mesenchymal transition
lnc-RNA: long non-coding RNA
NSCLC: Non-small cell lung cancer
PBS: phosphate buffered saline
PFS: progression-free survival
qRT-PCR: quantitative real-time PCR
ROC: receiver operating characteristic
siRNA: short interfering RNA
TNM: tumor node metastasis
