# Supplementary material for: Correlation of long non-coding RNA H19 expression with cisplatin-resistance and clinical outcome in lung adenocarcinoma
Source: Oncotarget. 2016 Nov 29;8(2):2558–67. doi: 10.18632/oncotarget.13708 (PMC5356823; doi:10.18632/oncotarget.13708)
Supplement: Supplementary file 1 [file oncotarget-08-2558-s001.pdf]

Correlation of long non-coding RNA *H19* expression with cisplatin-resistance and clinical outcome in lung adenocarcinoma

Supplementary Materials

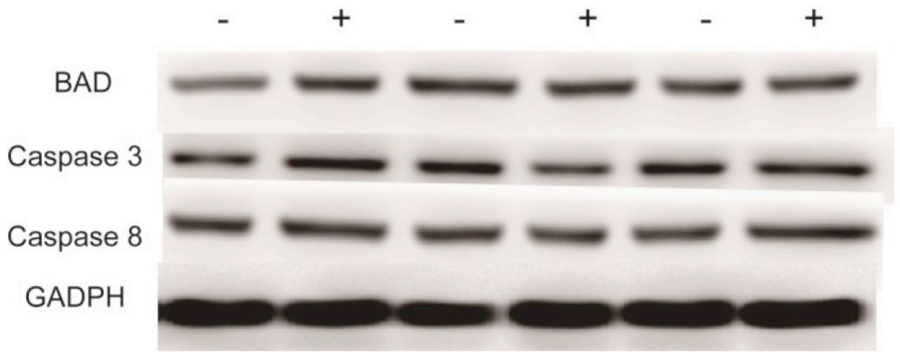

Supplementary Figure S1: Western blot analysis: BAD, Caspase3, Caspase 8. "+" indicates apoptosis markers detected with cisplatin, "−" indicates apoptosis detected without cisplatin.
